# Hepatitis E: Update on Prevention and Control

**DOI:** 10.1155/2018/5769201

**Published:** 2018-01-09

**Authors:** Juliana Gil Melgaço, Noemi Rovaris Gardinali, Vinicius da Motta de Mello, Mariana Leal, Lia Laura Lewis-Ximenez, Marcelo Alves Pinto

**Affiliations:** ^1^Ambulatório/Laboratório de Hepatites Virais, Instituto Oswaldo Cruz, Fundação Oswaldo Cruz, Rio de Janeiro, RJ, Brazil; ^2^Laboratório de Desenvolvimento Tecnológico em Virologia, Instituto Oswaldo Cruz, Fundação Oswaldo Cruz, Rio de Janeiro, RJ, Brazil

## Abstract

Hepatitis E virus (HEV) is a common etiology of acute viral hepatitis worldwide. Recombinant HEV vaccines have been developed, but only one is commercially available and licensed in China since 2011. Epidemiological studies have identified genotype 3 as the major cause of chronic infection in immunocompromised individuals. Ribavirin has been shown to be effective as a monotherapy to induce HEV clearance in chronic patients who have undergone solid organ transplant (SOT) under immunosuppressive therapy. Efforts and improvements in prevention and control have been made to reduce the instances of acute and chronic hepatitis E in endemic and nonendemic countries. However, this review shows that further studies are required to demonstrate the importance of preventive vaccination and treatment worldwide, with emphasis on hepatitis E infection in the public health system.

## 1. History of Hepatitis E

After the development of serological tests for the detection of hepatitis A and hepatitis B viruses in the 1980s, a large waterborne outbreak which occurred in 1955-1956 in New Delhi, India, was investigated and classified as enteric non-A, non-B hepatitis [1]. Same epidemiological investigation was performed in Kashimir, India, during a non-A, non-B hepatitis outbreak in 1978-1979 [2]. To both outbreaks, the laboratorial tests confirmed that hepatitis E virus (HEV) was the etiology agent [3, 4]. It was the first time that HEV was identified and associated with waterborne epidemics [3, 4].

Years later, in 1983, HEV was first identified by electron microscopy when they replicated the infection with a pool of human feces in one volunteer who had previously contacted with hepatitis A virus. It was also the first time to detect genotype 1 [4, 5].

Other cases occurred in Costa Rica in 1975 [2, 6]. In 1988, an outbreak in Somalia, Africa, was reported reaching 11,000 people [7]. In Latin America, the first detection of HEV was described in Mexico, during an outbreak between 1986 and 1987, with detection of genotype 2 [5, 8]. The largest epidemic described was in China between 1986 and 1988, presenting 120,000 people with the disease caused by genotype 1 [9].

HEV genome was first cloned in the early 1990s, and genotypes 3 and 4 were identified in 1995 and 2003, respectively [4, 6, 7]. Other genotypes, such as 5, 6, and 7, have already been documented in animals, and these are considered reservoirs of the virus [10, 11].

## 2. Hepatitis E Virus, Transmission, and Diagnosis

Hepatitis E virus (HEV) is a small, nonenveloped virus (27–34 nm) with icosahedral symmetry in the Hepeviridae Family. HEV is divided into two genera:* Orthohepevirus* with four species (A–D) and* Piscihepevirus* with one species (A) [6, 10, 12, 13].* Orthohepevirus A *species include almost all mammalian HEV variants; it is divided into seven genotypes, of which at least five of them are of interest in human public health. First, genotypes 1 and 2 (HEV-1 and HEV-2) infect only humans and are responsible for sporadic cases and large waterborne outbreaks in endemic areas. Furthermore, genotypes 3 and 4 (HEV-3 and HEV-4) are able to infect humans and animals and are enzootic in pigs, which are considered the main reservoir of HEV in the environment [4, 6, 10]. Until now, only one report of human HEV infection with genotype 7 (HEV-7) has been documented [14].

*Orthohepevirus B* species include viruses, which infect birds, while* Orthohepevirus C* include viruses that infect rat and ferret. Viruses isolates were detected in bats, and they were grouped into* Orthohepevirus D *species.* Piscihepevirus* genus is formed by only one species,* Piscihepevirus A*, and comprises all the isolates identified in trout fish [15].

Nevertheless, there is one serotype of hepatitis E circulating worldwide [4, 6, 10, 16, 17]. To our knowledge, Table 1 shows the genotypes detected in humans and animals, as well as geographic distribution in which the genome was recently detected and frequently circulating.

HEV contains a 7.2 kb single-stranded, positive-sense RNA genome that consists of three overlapping open reading frames (ORF) with distinct functions: ORF1, ORF2, and ORF3. ORF1 encodes a nonstructural polyprotein (with 1690 amino acids (aa)), which consists of proteins required for RNA replication such as methyltransferase, papain-like cysteine protease, RNA helicase, and RNA-dependent RNA polymerase. ORF2 is the most important structural protein for targeting HEV vaccine development since it encodes the capsid protein, which carries neutralizing epitopes inducing antibody production in the host and reservoirs. ORF3 has 123 aa, overlaps with ORF2, and encodes a small multifunctional protein involved in viral particle secretion [4, 6, 10, 17].

Complete particles of HEV are vulnerable to boiling or frying and they become inactivated after 5 minutes at temperatures above 90°C. To completely inactivate HEV in the food, an internal temperature of 71°C for 20 min is necessary [18]. HEV is also susceptible to chlorine disinfection in fomites and water supplies [4, 6, 19].

Hepatitis E is mainly transmitted by a fecal-oral route through contaminated water ingestion and the consumption of undercooked pork or wild boar (Table 1). However, transmission by blood components is increasingly recognized [20, 21]. Poor hygiene and untreated sewage are correlated with infections by the HEV-1 and HEV-2 genotypes in developing countries. Outbreaks and sporadic cases are related to the transiently contamination of water supplies. Instances of autochthonous hepatitis E by genotypes 3 and 4 are increasing in developed countries with high consumption of raw or uncooked pork meat since hepatitis E is categorized as a zoonotic disease with domestic animals as reservoir (mainly pigs) (Table 1) [4, 6, 10, 17].

The diagnosis of HEV infection is not easy considering the short period of HEV viremia, which is not always concomitant with the onset of symptoms. Serum samples are used to perform molecular detection of RNA and specific-antibodies against capsid (ORF2) as the main tool for HEV diagnosis. Two serological markers can be used to investigate the presence of past or recent HEV infection. The first is anti-HEV IgM, which indicates the acute phase, and the second is anti-HEV IgG, which indicates current infection when observed together with anti-HEV IgM detection or past contact when it is detected alone [13, 16, 22, 23]. RNA testing is usually useful when serology is difficult to interpret due to cross-reactivity with polyclonal antibody immune response and in immunocompromised patients, in which the antibody response may be undetectable. Nevertheless, the technique is vital for genotyping for epidemiological purposes [24].

## 3. Epidemiology

**Figure 1:**
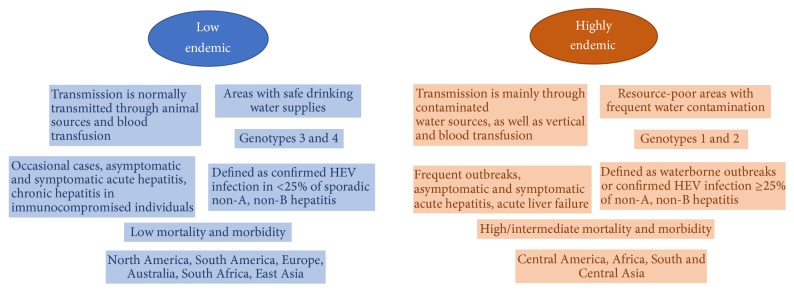
Characteristics of two distinct epidemiological patterns of hepatitis E infection [28, 29].

According to the World Health Organization (WHO), HEV infection is one of the most common causes of acute hepatitis and has a large distribution worldwide [25]. It is estimated that 2.3 billion people have already been infected with HEV, and 70,000 deaths are attributed to HEV annually [26, 27]. Two distinct epidemiological patterns have been observed in different regions of the globe. These patterns seem to be correlated with the distribution of HEV genotypes, transmission routes, source of virus infection, disease prevalence, and, in some cases, clinical characteristics of the disease. The epidemiology and clinical features of HEV infection are primarily determined by the predominant genotype in the region and their respective hosts (Table 1). Characteristics of these two faces of hepatitis E infection correlated with geographic distribution are summarized in Figure 1 according to Center of Diseases Control and World Health Organization [28, 29].

HEV genotypes 1 and 2 are highly endemic in tropical and subtropical areas where hepatitis E occurs as outbreaks and sporadic cases transmitted by a fecal-oral route through contaminated water caused by genotypes 1 or 2 [30]. Outbreaks and sporadic cases can occur globally, but there are distinct circulations of HEV genotypes, as in Asia and Africa, where genotypes 1 and 2 are predominant, respectively (Table 1, Figure 1). Additionally, genotypes 3 and 4 are also frequently detected in Asia [29, 31–33] (Table 1, Figure 1). The prevalence of antibodies against HEV (anti-HEV IgG) in those regions is between 3 and 27% [29, 34].

Outbreaks of HEV-1 and HEV-2 have been documented in areas with limited access to water and inadequate sanitary conditions, generally in resource-limited countries [25] (Figure 1). The WHO showed that African and Asia are the areas most affected by infection of these genotypes. The prevalence of anti-HEV IgG in Africa ranges from 4.6 to 10.7% in the general population [29, 35]. Asia shows a higher prevalence than Africa with anti-HEV IgG frequencies that can reach 34.8% to 94% [29, 34, 36–38].

Outbreaks of HEV-1 and 2 reach thousands of people, and up to 15% of the infected population present signals and symptoms [39, 40]. Overall morbidity rates are higher among teenagers and young adults (between 10 and 40 years old) but lower in children and elderly people [41]. High morbidity and severity have been observed among pregnant women and patients with preexisting chronic liver disease, leading to fulminant hepatitis [42, 43]. The clinical disease is characterized by acute, self-limited hepatitis that is clinically and biochemically indistinguishable from other types of viral hepatitis [44] (Figure 1).

Besides fecal-oral transmission, vertical [45, 46] and parenteral [47] routes of HEV-1 and 2 transmission are also recognized. HEV infection with genotypes 1 frequently determines symptomatic disease in pregnant women that can be severe, especially in the third trimester determining acute liver failure (ALF) with a mortality rate of 15–25% [48, 49]. Infection with HEV-1 during pregnancy is also related to an increased risk of adverse outcomes of pregnancy as spontaneous abortion, fetal death in utero, and premature delivery in patients with icteric hepatitis or with ALF caused by HEV [50, 51]. In addition, vertical transmission can result in complications to the fetuses and neonates, such as anicteric or icteric hepatitis hypoglycemia and neonatal death [49, 50]. High mortality in pregnant women is mostly observed in outbreaks of hepatitis E in India, and findings showed that elevated viral load as well as hormones levels alterations, immunological changes, and poor nutrition during pregnancy have been correlated with worst outcome, which makes women more susceptible [49–53].

Regions with adequate sanitary conditions and well-controlled water supplies are considered low endemic areas for hepatitis E, such as Europe, East Asia (including China), and the Americas (Figure 1). In these regions, the disease is less frequent and occurs as sporadic cases [13, 29, 54, 55], in which anti-HEV prevalence is 7% to 10% [29, 54, 55]. Autochthonous cases of HEV infection in these areas appear to be associated with occasional zoonotic transmission by genotypes 3 and 4 from domestic animals (most often from pigs to humans). This transmission can occur through the ingestion of raw or undercooked food containing the virus, especially swine products [56].

Additionally HEV (especially genotype 3 in Europe) can be iatrogenically transmitted between humans through infected blood and blood products [57]. Transfusion-associated HEV infections have been reported also in Japan (for genotypes 3 and 4) and China (for genotypes 1 and 4), although this pathway is less common than the zoonotic and the waterborne infections [58, 59].

The prevalence rates of anti-HEV in Europe could be explained by pork consumption, as in France and Germany, where the seroprevalence is 17% and 35%, respectively. In France, anti-HEV IgG prevalence was also high in an investigation of blood banks (52%), which reinforces the findings about the prevalence of hepatitis E in Europe. Recent studies detected genotypes 1, 3, and 4 in Europe [29, 60–63]. In low endemic areas, HEV-1 is usually detected in travelers coming from endemic regions [29, 60–63].

In the Americas, the seroprevalence of anti-HEV IgG ranges from 3 to 31%, which can be explained by the sporadic HEV cases reported in the area [29, 64, 65]. Genotypes 1 through 3 have been documented in humans and animals in Uruguay (HEV-1 and HEV-3), Colombia (HEV-3), Argentina (HEV-3), Mexico (HEV-2, only humans), Venezuela (HEV-1 and HEV-3), Brazil (HEV-3), and the United States of America (HEV-3) [5, 8, 10, 29, 64–70].

Conventionally, symptomatic HEV-3 and HEV-4 infection results in self-limited acute hepatitis in humans. However, in recent years, the occurrence of chronic hepatitis by infection, especially with genotype 3, has been described in immunosuppressed patients, especially posttransplant patients [71–73]. The transfusion of blood and blood products, solid organ transplantation (SOT), stem cells, and pork-derived products constitute the principal sources of infection, especially for immunosuppressed individuals. However, they can be avoided by screening biological samples in blood banks [74, 75]. Besides, patients with preexisting chronic liver disease and those infected with HEV-3 can progress to fulminant hepatitis, as observed with genotype 1 [76].

Acute or chronic HEV infection caused by genotypes 1–4 may also cause extrahepatic manifestations that include neurological disorders, kidney injury, acute pancreatitis, and hematological abnormalities. Particularly for HEV infection, physicians need to have attention to neurological manifestations such as Guillain-Barré syndrome, brachial neuritis, and meningoencephalitis, which are a risk, despite rare occurrence in viral hepatitis [77].

## 4. Hepatitis E Vaccination

The need for hepatitis E vaccine is related to its worldwide distribution. The WHO estimates that 44,000 deaths were caused by HEV in 2015, representing 3.3% of the mortality due to viral hepatitis [29]. Normally, pregnant women with HEV-1 infection have the worst outcome and have been considered the main target group to receive vaccinations [35, 52, 78, 79]. Public health surveillance in pregnant and nonpregnant population is also extremely important for controlling the number of outbreaks, along with improving sanitation in endemic and nonendemic countries [13, 31, 34, 54, 60, 62, 63, 80, 81].

It is not feasible to develop live attenuated or inactivated vaccines for hepatitis E virus by* in vitro* cell culture replication [82–85]. Passive immunoprophylaxis has not succeeded in preventing infection, but only the symptoms of hepatitis. On the other hand, active immunization has been demonstrated to be effective in experimental animal models [86]. Thus, several studies have focused on the development of recombinant vaccines [82–85].

The only vaccine that is commercially available is the HEV 239 vaccine (Hecolin, Xiamen Innovax Biotech, China), which has been registered in China since 2011. However, it has not yet been approved in other countries [87, 88]. This recombinant vaccine which contains 26 aa and is an extension from the N terminal of another peptide, E2, from the HEV capsid protein, which is the one major structural protein. This approach for the vaccine is possible because HEV is antigenically conserved, presenting only one identified serotype, which was observed to be protective for all four HEV genotypes (HEV-1, HEV-2, HEV-3, and HEV-4) [87, 89–91].

The HEV 239 vaccine is protective against hepatitis in animal models based on Rhesus monkeys immunized and challenged with infectious virus strains (genotypes 1 and 4) [83, 85]. The vaccine was found to induce effective neutralizing antibodies against HEV [83, 92, 93]. In mouse models, a strong T cell-dependent antibody response was observed after vaccination, which was partly attributed to two T cell epitopes located in the portion of aa 533–552 on the HEV capsid peptide [6, 82, 94].

The vaccination schedule in China with HEV 239 vaccine involves three doses administered intramuscularly at months 0, 1, and 6. In 2010, Zhu and colleagues performed a phase 3 trial in healthy adults (>16 years old) in China using the HEV 239 vaccine, which contained 30 *μ*g of the purified antigen plus aluminum hydroxide as adjuvant. The vaccine's efficacy is greater than 90% for 1 year after one dose and for 4.5 years after three doses [94–96]. Additionally, the HEV 239 vaccine is safe for both pregnant women and the fetus [90].

Considering that only China has experience with vaccination against hepatitis E, and HEV infection has remained a health problem, the cost-effectiveness of the HEV vaccine has been debated. The Hecolin HEV 239 vaccine from Xiamen Innovax Biotech costs around USD 17.60–41.70 per dose [78, 88], which is less expensive than hepatitis A vaccine (median price: USD 23.21 per dose) [97]. Consequently, as immunization can reduce the cost of hospitalization and treatment, the implementation of hepatitis E vaccine could be a cost-effective health intervention at the market vaccine price. In addition, given the budget limitations in developing countries, the single-dose schedule would be more realistic to apply and could influence the adaption of immunization policies in highly endemic and low endemic countries.

Nevertheless, further studies are necessary to establish or improve HEV immunization worldwide in humans and animals (e.g., domestic pigs). Furthermore, health surveillance will be very important for monitoring the prevalence and incidence of HEV before and after immunization. In addition to these efforts, developing countries still need sanitary and environmental improvements. For more clarification, Table 2 briefly summarizes the ways of preventing hepatitis E nowadays.

## 5. Treatment

Treatment for hepatitis E infection can be justified by the chronic and persistent infections commonly caused by genotype 3 and involved with immunosuppressive or immunocompromised conditions [32, 98–101]. SOT, HIV, and hemodialysis patients are populations that are at risk for chronic hepatitis E [32, 98]. Several studies describe that HEV clearance was achieved by administering decreasing doses of immunosuppressants that target T-cells after SOT [98, 102, 103]. Therapies using ribavirin and pegylated-interferon succeeded in establishing a sustained virologic response after 3–6 months of treatment after SOT, with patients presenting a restoration of lymphocyte count [98].

**Figure 2:**
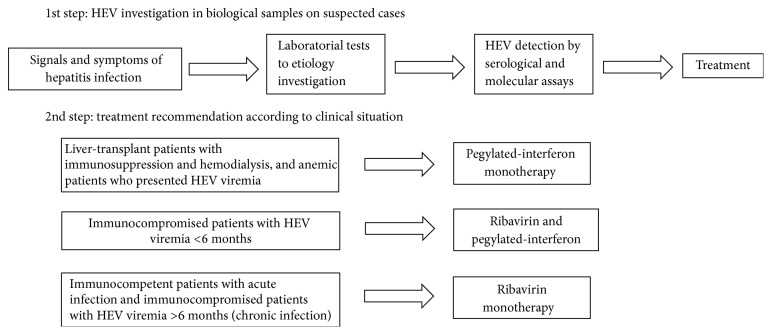
Update of HEV infection treatment recommendations.

Recently, a large follow-up study related the effect of ribavirin as a monotherapy for SOT recipients with prolonged HEV viremia. Kamar and colleagues observed HEV clearance in 95% of patients on ribavirin monotherapy with a median dose of 600 mg/day for 3–6 months. The only side effect in this study was anemia, which required reduction of the ribavirin dose in 29% of the patients, as well as the use of erythropoietin (54%) and blood transfusion (12%) [32, 98]. Pegylated-interferon had a successful effect on liver-transplant patients with immunosuppression and hemodialysis who presented HEV viremia. However, it is not indicated in other types of SOT, such heart, lung, and kidney SOT, since it can increase the risk of organ rejection [98, 100]. Thus, the recent findings suggest that ribavirin is an antiviral therapy to treat HEV chronic infection in immunocompromised patients.  Figure 2 shows the therapies normally used to treat hepatitis E infection according to patient's clinical situation in recent studies.

HEV replication is inhibited by the inactivation RNA polymerase function using antiviral drugs such as zinc salt and nucleoside analogue 2′-C-methylcytidine (2CMC) in human cell lines* in vitro*. Thus, they have been suggested as potential drugs to control HEV infection [17, 103]. When 2CMC is administered with ribavirin, the activity and efficacy of ribavirin are compromised [103]. Other drugs have also been tested* in vitro*, such as sofosbuvir, calcineurin inhibitors, and mTOR inhibitors, which may help in virus clearance and show promise for future treatments [6, 17, 103].

There is few information about treatments against acute hepatitis E infection, for which ribavirin is recommended to treat immunocompetent and immunocompromised individuals [6, 99, 102]. Nevertheless, safe and effective treatment has been achieved with 3-4 weeks of ribavirin treatment, showing the reestablishment of liver enzymes levels in immunocompetent patients who usually need symptomatic treatment due to the short duration of HEV viremia, as well as immunocompromised subjects who are characterized by elevated transaminase levels with HEV viremia in the acute phase [6, 102].

## 6. Conclusion

Several studies have been performed to investigate HEV infection and reduce the numbers of hepatitis cases caused by this etiology. Considerable improvements in vaccination and treatment have been achieved, but there have been limitations that bound the success of HEV elimination. This is particularly true in developing countries, where HEV remains an important health problem. In this review, we have presented an update on the cost-effectiveness vaccination to spread the program worldwide. Together with information about treatment of immunocompromised and immunocompetent individuals, approaches were suggested to help continue the surveillance and optimize future research in this area.

## Figures and Tables

**Table 1:** HEV genotypes in human and animals hosts and their geographical distribution.

Genotypes	Host species	Geographical distribution	References
1	Human	Asia, Africa, Central America	Nelson et al., 2016 [104]; Kamar et al., 2014 [32]; Kamar et al., 2012 [105]
2	Human	West Africa, Mexico	Nelson et al., 2016 [104]; Kamar et al., 2014 [32]; Kamar et al., 2012 [105]
3	Human, pig, wild boar, red deer, mongoose, rabbit	Europe, East Asia, South Africa, and Americas	Pinto et al., 2017 [5] Nelson et al., 2016 [104]; Kamar et al., 2014 [32]; Kamar et al., 2012 [105];
4	Human, pig, sheep, cattle	Asia, and Europe	Pinto et al., 2017 [5] Nelson et al., 2016 [104]; Kamar et al., 2014 [32]; Kamar et al., 2012 [105]; Wang and Ma, 2010 [106];
5	Wild boar	Asia	Pinto et al., 2017 [5] Sridhar et al., 2017 [107]; Smith et al., 2015 [15]; Kamar et al., 2014 [32]
6	Wild boar	Asia	Pinto et al., 2017 [5] Sridhar et al., 2017 [107]; Smith et al., 2015 [15]; Kamar et al., 2014 [32]
7	Dromedary camel	Asia	Woo et al., 2014 [11] Sridhar et al., 2017 [107]

**Table 2:** Preventive measures for hepatitis E in 2010 [4, 18, 19, 29, 104, 108].

Preventive action	Procedures
Virus inactivation	Water and food to ingest: boiling or frying at temperatures above 90°C, wash fruit and vegetables with chlorine solutions; fomites and water supplies: chlorine disinfection.
Sanitation, hygiene, and surveillance	Community: treat sewage and water supplies. Personal: wash hands; use gloves to prepare food. Healthcare workers: wear individual biosafety clothes during outbreaks, and in blood procedure manipulation; perform laboratorial screening in the blood bank.
Vaccination	Perform mass vaccination with a safety vaccine to susceptible people and animals.

## References

[1] Viswanathan R. (1957). Infectious hepatitis in Delhi (1955-56): a critical study-epidemiology 1957. *The National Medical Journal of India*.

[2] Khuroo M. S. (1980). Study of an epidemic of non-A, non-B hepatitis. Possibility of another human hepatitis virus distinct from post-transfusion non-A, non-B type. *American Journal of Medicine*.

[3] Wong D. C., Purcell R. H., Sreenivasan M. A., Prasad S. R., Pavri K. M. (1980). Epidemic and endemic hepatitis in India: evidence for a non-A, non-B hepatitis virus aetiology. *The Lancet*.

[4] Teshale E., Hu D. (2011). Hepatitis E: epidemiology and prevention. *World Journal of Hepatology*.

[5] Pinto M., de Oliveira J., González J. (2017). Hepatitis A and E in South America: New Challenges Toward Prevention and Control. *Human Virology in Latin America-From Biology to Control*.

[6] Khuroo M. S., Khuroo M. S., Khuroo N. S. (2016). Hepatitis E: discovery, global impact, control and cure. *World Journal of Gastroenterology*.

[7] Mushahwar I. K. (2008). Hepatitis E virus: molecular virology, clinical features, diagnosis, transmission, epidemiology, and prevention. *Journal of Medical Virology*.

[8] Rendon J., Hoyos M. C., Di Filippo D. (2016). Hepatitis E virus genotype 3 in Colombia: survey in patients with clinical diagnosis of viral hepatitis. *PLoS ONE*.

[9] Aye T. T., Uchida T., Ma X.-Z. (1992). Complete nucleotide sequence of a hepatitis e virus isolated from the xinjiang epidemic (1986–1988) of china. *Nucleic Acids Research*.

[10] Gardinali N. R., Guimarães J. R., Melgaço J. G. (2017). Cynomolgus monkeys are successfully and persistently infected with hepatitis E virus genotype 3 (HEV-3) after long-term immunosuppressive therapy. *PLoS ONE*.

[11] Woo P. C. Y., Lau S. K. P., Teng J. L. L. (2014). New hepatitis E virus genotype in camels, the Middle East. *Emerging Infectious Diseases*.

[12] FitzSimons D., Hendrickx G., Vorsters A., Van Damme P. (2010). Hepatitis A and E: update on prevention and epidemiology. *Vaccine*.

[13] Domanović D., Tedder R., Blümel J. (2017). Hepatitis E and blood donation safety in selected European countries: a shift to screening?. *Eurosurveillance*.

[14] Lee G.-H., Tan B.-H., Teo E. C. (2016). Chronic infection with camelid hepatitis E virus in a liver transplant recipient who regularly consumes camel meat and milk. *Gastroenterology*.

[15] Smith D. B., Simmonds P., Jameel S. (2015). Consensus proposals for classification of the family Hepeviridae. *Journal of General Virology*.

[16] De Almeida Ramos D., Miani M., Pandolfi R. (2016). Production and characterization of a Brazilian candidate antigen for Hepatitis E Virus genotype 3 diagnosis. *FEMS Microbiology Letters*.

[17] Kaushik N., Subramani C., Anang S., Muthumohan R. (2017). Zinc salts block hepatitis E virus replication by inhibiting the activity of viral rna-dependent rna polymerase. *Journal of Virology*.

[18] Barnaud E., Rogée S., Garry P., Rose N., Pavio N. (2012). Thermal inactivation of infectious hepatitis E virus in experimentally contaminated food. *Applied and Environmental Microbiology*.

[19] Schielke A., Filter M., Appel B., Johne R. (2011). Thermal stability of hepatitis e virus assessed by a molecular biological approach. *Virology Journal*.

[20] Colson P. Transfusion-associated hepatitis E, France.

[21] Hewitt P. E. Hepatitis E virus in blood components: a prevalence and transmission study in southeast England.

[22] Zhang J., Zhang X.-F., Zhou C. (2014). Protection against hepatitis E virus infection by naturally acquired and vaccine-induced immunity. *Clinical Microbiology and Infection*.

[23] Inagaki Y., Oshiro Y., Tanaka T. (2015). A Nationwide Survey of Hepatitis E Virus Infection and Chronic Hepatitis E in Liver Transplant Recipients in Japan. *EBioMedicine*.

[24] Zhao C., Wang Y. (2016). Laboratory diagnosis of HEV infection. *Advances in Experimental Medicine and Biology*.

[25] R. A. The Global Prevalence of Hepatitis E Virus Infection and Susceptibility: A Systematic Review, World Health Organization, Department of Immunization, Vaccines and Biologicals. http://apps.who.int/iris/bitstream/10665/70513/1/WHO_IVB_10.14_eng.pdf.

[26] Rein D. B., Stevens G. A., Theaker J., Wittenborn J. S., Wiersma S. T. (2012). The global burden of hepatitis E virus genotypes 1 and 2 in 2005. *Hepatology*.

[27] Mirazo S., Ramos N., Mainardi V., Gerona S., Arbiza J. (2014). Transmission, diagnosis, and management of hepatitis E: an update. *Hepatic Medicine: Evidence and Research*.

[28] Teshale E. H. (2017). *Travelers' Health, Hepatitis E, Chapter 3, Yellow Book*.

[29] Who (2017). *Hepatitis E*.

[30] Yugo D. M., Meng X.-J. (2013). Hepatitis E virus: foodborne, waterborne and zoonotic transmission. *International Journal of Environmental Research and Public Health*.

[31] Dai X., Dong C., Zhou Z. (2013). Hepatitis E virus genotype 4, Nanjing, China, 2001–2011. *Emerging Infectious Diseases*.

[32] Kamar N., Dalton H. R., Abravanel F., Izopet J. (2014). Hepatitis E virus infection. *Clinical Microbiology Reviews*.

[33] Minagi T., Okamoto H., Ikegawa M. (2016). Hepatitis E virus in donor plasma collected in Japan. *Vox Sanguinis*.

[34] Ren F., Zhao C., Wang L. (2014). Hepatitis E virus seroprevalence and molecular study among blood donors in China. *Transfusion*.

[35] Goumba C. M., Yandoko-Nakouné E. R., Komas N. P. (2010). A fatal case of acute hepatitis e among pregnant women, Central African Republic. *BMC Research Notes*.

[36] You S., Rong Y., Zhu B. (2013). Changing etiology of liver failure in 3,916 patients from northern China: a 10-year survey. *Hepatology International*.

[37] Izopet J., Labrique A. B., Basnyat B. (2015). Hepatitis E virus seroprevalence in three hyperendemic areas: Nepal, Bangladesh and southwest France. *Journal of Clinical Virology*.

[38] Kang Y.-H., Cong W., Zhang X.-Y., Wang C.-F., Shan X.-F., Qian A.-D. (2017). Hepatitis E virus seroprevalence among farmers, veterinarians and control subjects in Jilin province, Shandong province and Inner Mongolia Autonomous Region, China. *Journal of Medical Virology*.

[39] Goel A., Aggarwal R. Advances in hepatitis E–II: epidemiology, clinical manifestations, treatment and prevention.

[40] Naik S. R. A large waterborne viral hepatitis E epidemic in Kanpur, India.

[41] R A. Clinical presentation of hepatitis E. - NCBI.

[42] Khuroo M. S. Incidence and severity of viral hepatitis in pregnancy.

[43] Kumar Acharya S., Kumar Sharma P., Singh R. (2007). Hepatitis E virus (HEV) infection in patients with cirrhosis is associated with rapid decompensation and death. *Journal of Hepatology*.

[44] Singh A., Seth R., Gupta A., Nayak B., Acharya S. k. (2016). Chronic hepatitis E – an emerging disease in an immunocompromised host. *Gastroenterology Report*.

[45] Khuroo M. S., Kamali S., Jameel S. (1995). Vertical transmission of hepatitis E virus. *The Lancet*.

[46] Kumar R. M., Uduman S., Rana S., Kochiyil J. K., Usmani A., Thomas L. (2001). Sero-prevalence and mother-to-infant transmission of hepatitis E virus among pregnant women in the United Arab Emirates. *European Journal of Obstetrics & Gynecology and Reproductive Biology*.

[47] Arankalle V. A., Chobe L. P. (1999). Hepatitis E virus: can it be transmitted parenterally?. *Journal of Viral Hepatitis*.

[48] Naidu S. S., VIswanathan R. (1957). Infectious hepatitis in pregnancy during Delhi epidemic. *Indian Journal of Medical Research*.

[49] Sharma S., Kumar A., Kar P. (2017). Risk factors for vertical transmission of hepatitis E virus infection. *Journal of Viral Hepatitis*.

[50] Jilani N., Das B. C., Husain S. A. (2007). Hepatitis E virus infection and fulminant hepatic failure during pregnancy. *Journal of Gastroenterology and Hepatology*.

[51] Salam G. D., Kumar A., Kar P., Aggarwal S., Husain A., Sharma S. (2013). Serum tumor necrosis factor-alpha level in hepatitis E virus-related acute viral hepatitis and fulminant hepatic failure in pregnant women. *Hepatology Research*.

[52] Kumar A., Devi S. G., Kar P. (2014). Association of cytokines in hepatitis E with pregnancy outcome. *Cytokine*.

[53] Bose P. D., Das B. C., Kumar A., Gondal R., Kumar D., Kar P. (2011). High viral load and deregulation of the progesterone receptor signaling pathway: association with Hepatitis E-related poor pregnancy outcome. *Journal of Hepatology*.

[54] Fierro N. A., Realpe M., Meraz-Medina T., Roman S., Panduro A. (2016). Hepatitis e virus: an ancient hidden enemy in Latin America. *World Journal of Gastroenterology*.

[55] Panduro A., Meléndez G. E., Fierro N. A., Madrigal B. R., Zepeda-Carrillo E. A., Román S. (2011). Epidemiology of viral hepatitis in Mexico. *Salud Pública de México*.

[56] Dalton H. R., Kamar N., Izopet J. (2014). Hepatitis E in developed countries: current status and future perspectives. *Future Microbiology*.

[57] Hewitt P. E., Ijaz S., Brailsford S. R. (2014). Hepatitis e virus in blood components: a prevalence and transmission study in southeast England. *The Lancet*.

[58] Satake M., Matsubayashi K., Hoshi Y. (2017). Unique clinical courses of transfusion-transmitted hepatitis E in patients with immunosuppression. *Transfusion*.

[59] Zhang L., Jiao S., Yang Z. (2017). Prevalence of hepatitis E virus infection among blood donors in mainland China: a meta-analysis. *Transfusion*.

[60] Clemente-Casares P., Rodriguez-Manzano J., Girones R. (2009). Hepatitis E virus genotype 3 and sporadically also genotype 1 circulate in the population of Catalonia, Spain. *Journal of Water and Health*.

[61] Riveiro-Barciela M., Rodríguez-Frías F., Buti M. (2013). Hepatitis E virus: new faces of an old infection. *Annals of Hepatology*.

[62] Lapa D., Capobianchi M. R., Garbuglia A. R. (2015). Epidemiology of hepatitis E virus in European countries. *International Journal of Molecular Sciences*.

[63] Bouamra Y., Gérolami R., Arzouni J.-P. (2013). Emergence of autochthonous infections with hepatitis E virus of genotype 4 in Europe. *Intervirology*.

[64] Lopes dos Santos D. R., Lewis-Ximenez L. L., da Silva M. F. M., de Sousa P. S. F., Gaspar A. M. C., Pinto M. A. (2010). First report of a human autochthonous hepatitis E virus infection in Brazil. *Journal of Clinical Virology*.

[65] Mirazo S., Ramos N., Russi J. C., Gagliano G., Arbiza J. (2011). Detection and molecular characterization of sporadic cases of acute human hepatitis E virus infection in Uruguay. *Archives of Virology*.

[66] García C. G., Sánchez D., Villalba M. C. M. (2012). Molecular characterization of hepatitis E virus in patients with acute hepatitis in Venezuela. *Journal of Medical Virology*.

[67] Martínez Wassaf M. G., Pisano M. B., Barril P. A. (2014). First detection of hepatitis E virus in Central Argentina: Environmental and serological survey. *Journal of Clinical Virology*.

[68] De Souza A. J. S., Gomes-Gouvêa M. S., Soares M. D. C. P. (2012). HEV infection in swine from Eastern Brazilian Amazon: evidence of co-infection by different subtypes. *Comparative Immunology, Microbiology and Infectious Diseases*.

[69] Passos-Castilho A. M., de Sena A., Geraldo A., Spada C., Granato C. F. H. (2016). High prevalence of hepatitis E virus antibodies among blood donors in Southern Brazil. *Journal of Medical Virology*.

[70] Mirazoa S., Gardinalib N. R., D'Alboraa C. (2018). Serological and virological survey of hepatitis E virus (HEV) in animal reservoirs from Uruguay reveals elevated prevalences and a very close phylogenetic relationship between swine and human strains. *Veterinary Microbiology*.

[71] Gérolami R., Moal V., Colson P. (2008). Chronic hepatitis E with cirrhosis in a kidney-transplant recipient. *The New England Journal of Medicine*.

[72] Kamar N., Selves J., Mansuy J.-M. (2008). Hepatitis E virus and chronic hepatitis in organ-transplant recipients. *The New England Journal of Medicine*.

[73] Geng Y., Zhang H., Huang W. (2014). Persistent hepatitis E virus genotype 4 infection in a child with acute lymphoblastic leukemia. *Hepatitis Monthly*.

[74] Schlosser B., Stein A., Neuhaus R. (2012). Liver transplant from a donor with occult HEV infection induced chronic hepatitis and cirrhosis in the recipient. *Journal of Hepatology*.

[75] Koenecke C., Pischke S., Beutel G. (2014). Hepatitis e virus infection in a hematopoietic stem cell donor. *Bone Marrow Transplantation*.

[76] Dalton H., Hazeldine S., Banks M., Ijaz S., Bendall R. (2007). Locally acquired hepatitis E in chronic liver disease. *The Lancet*.

[77] Kamar N., Marion O., Abravanel F., Izopet J., Dalton H. R. (2016). Extrahepatic manifestations of hepatitis E virus. *Liver International*.

[78] Zhao Y., Zhang X., Zhu F., Jin H., Wang B. (2016). A preliminary cost-effectiveness analysis of hepatitis E vaccination among pregnant women in epidemic regions. *Human Vaccines & Immunotherapeutics*.

[79] Wang X. Prophylaxis against hepatitis E: at risk populations and human vaccines.

[80] Bortoliero A. L., Bonametti A. M., Morimoto H. K., Matsuo T., Reiche E. M. V. (2006). Seroprevalence for hepatitis E virus (HEV) infection among volunteer blood donors of the Regional Blood Bank of Londrina, State of Paraná, Brazil. *Revista do Instituto de Medicina Tropical de São Paulo*.

[81] El Sayed Z. M., Othman W. (2011). Role of hepatitis E infection in acute on chronic liver failure in Egyptian patients. *Liver International*.

[82] Kamili S. (2011). Toward the development of a hepatitis E vaccine. *Virus Research*.

[83] Zhang J., Liu C.-B., Li R.-C. (2009). Randomized-controlled phase II clinical trial of a bacterially expressed recombinant hepatitis E vaccine. *Vaccine*.

[84] Trabelsi K., Kamen A., Kallel H. (2014). Development of a vectored vaccine against Hepatitis E virus. *Vaccine*.

[85] Li S.-W., Zhao Q., Wu T., Chen S., Zhang J., Xia N.-S. (2015). The development of a recombinant hepatitis E vaccine HEV 239. *Human Vaccines & Immunotherapeutics*.

[86] Tsarev S. A., Tsareva T. S., Emerson S. U. (1994). Successful passive and active immunization of cynomolgus monkeys against hepatitis E. *Proceedings of the National Acadamy of Sciences of the United States of America*.

[87] Park S. B. (2012). Hepatitis e vaccine debuts. *Nature*.

[88] Riedmann E. M. (2012). Chinese biotech partnership brings first hepatitis E vaccine to the market.. *Human vaccines & immunotherapeutics*.

[89] Hepatitis E. (2015). vaccine: WHO position paper. *Wkly Epidemiol Rec*.

[90] Wu T., Zhu F.-C., Huang S.-J. (2012). Safety of the hepatitis E vaccine for pregnant women: a preliminary analysis. *Hepatology*.

[91] Wu X., Chen P., Lin H., Hao X., Liang Z. (2016). Hepatitis E virus: Current epidemiology and vaccine. *Human Vaccines and Immunotherapeutics*.

[92] Cheng X., Wang S., Dai X. (2012). Rabbit as a novel animal model for hepatitis E virus infection and vaccine evaluation. *PLoS ONE*.

[93] Wen J. Immunogenicity difference between two hepatitis E vaccines derived from genotype 1 and 4.

[94] Zhu F.-C., Zhang J., Zhang X.-F. (2010). Efficacy and safety of a recombinant hepatitis e vaccine in healthy adults: a large-scale, randomised, double-blind placebo-controlled, phase 3 trial. *The Lancet*.

[95] Zhang J., Zhang X., Huang S. (2015). Long-term efficacy of a hepatitis E vaccine. *The New England Journal of Medicine*.

[96] Su Y., Huang S., Guo M. (2017). Persistence of antibodies acquired by natural hepatitis E virus infection and effects of vaccination. *Clinical Microbiology and Infection*.

[97] Review of vaccine price data: submitted by WHO European Region Member States through the WHO/UNICEF Joint Reporting Form for 2013 (2015), 2017

[98] Kamar N., Lhomme S., Abravanel F. (2015). An early viral response predicts the virological response to ribavirin in hepatitis e virus organ transplant patients. *Transplantation*.

[99] Pischke S., Hardtke S., Bode U. (2013). Ribavirin treatment of acute and chronic hepatitis E: a single-centre experience. *Liver International*.

[100] Pischke S., Greer M., Hardtke S. (2014). Course and treatment of chronic hepatitis E virus infection in lung transplant recipients. *Transplant Infectious Disease*.

[101] Miyoshi M., Kakinuma S., Tanabe Y. (2016). Chronic hepatitis E infection in a persistently immunosuppressed patient unable to be eliminated after ribavirin therapy. *Internal Medicine*.

[102] Péron J. M., Abravanel F., Guillaume M. (2016). Treatment of autochthonous acute hepatitis E with short-term ribavirin: a multicenter retrospective study. *Liver International*.

[103] Qu C., Xu L., Yin Y., Peppelenbosch M. P., Pan Q., Wang W. (2017). Nucleoside analogue 2’-C-methylcytidine inhibits hepatitis E virus replication but antagonizes ribavirin. *Archives of Virology*.

[104] Nelson K. E., Heaney C. D., Labrique A. B., Kmush B. L., Krain L. J. (2016). Hepatitis E: prevention and treatment. *Current Opinion in Infectious Diseases*.

[105] Kamar N., Bendall R., Legrand-Abravanel F. (2012). Hepatitis E. *The Lancet*.

[106] Wang Y., Ma X. (2010). Detection and sequences analysis of sheep hepatitis E virus RNA in Xinjiang autonomous region. *Acta Microbiologica Sinica*.

[107] Sridhar S., Teng J., Chiu T., Lau S., Woo P. (2017). Hepatitis E virus genotypes and evolution: emergence of camel hepatitis E variants. *International Journal of Molecular Sciences*.

[108] Wu X., Chen P., Lin H., Hao X., Liang Z. (2016). Hepatitis E virus: current epidemiology and vaccine. *Human Vaccines and Immunotherapeutics*.

